# P-1249. Effect of Starting Inoculum and β-lactamase Retention on the Activity of Imipenem/Relebactam (I/R) and Aztreonam (ATM) plus I/R in Hollow Fiber Infection (HFIM) Studies of P. aeruginosa (PSA)

**DOI:** 10.1093/ofid/ofaf695.1440

**Published:** 2026-01-11

**Authors:** J Nicholas O’Donnell, Nicole L Shakerley, Kelly E Moolick, Avery I Nahorniak, Abimael Marrero, Katherine Young, Thomas Lodise

**Affiliations:** Albany College of Pharmacy and Health Sciences, Albany, NY; Albany College of Pharmacy and Health Sciences, Albany, NY; Albany College of Pharmacy and Health Sciences, Albany, NY; ACPHS, Albany, New York; Albany College of Pharmacy and Health Sciences, Albany, NY; Merck, Rahway, New Jersey; Albany College of Pharmacy and Health Sciences, Stratton, VA, United States, Stratton, VA

## Abstract

**Background:**

HFIMs are the gold standard pre-clinical PK/PD model for studying humanized antibiotic exposure-response relationships. Standard HFIM cartridges (Fibercell C2011) use 20 kD MWCO fibers, which retain β-lactamases from lysed bacteria. Newer cartridges (Fibercell C7011) have larger 0.03 µm pores and are less prone to β-lactamase retention. We evaluated whether bacterial killing differs between I/R and I/R + ATM in PSA HFIM studies using C2011 vs. C7011 cartridges.

**Figure 1.**
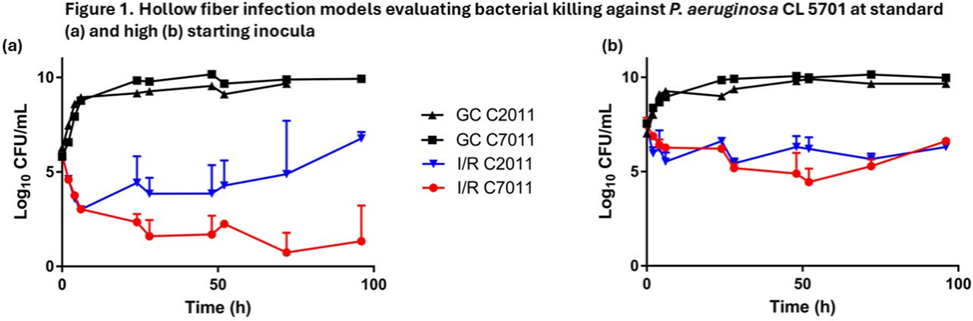
Hollow fiber infection models evaluating bacterial killing against P. aeruginosa CL 5701 at standard (a) and high (b) starting inocula I/R, imipenem/relebactam

**Figure 2.**
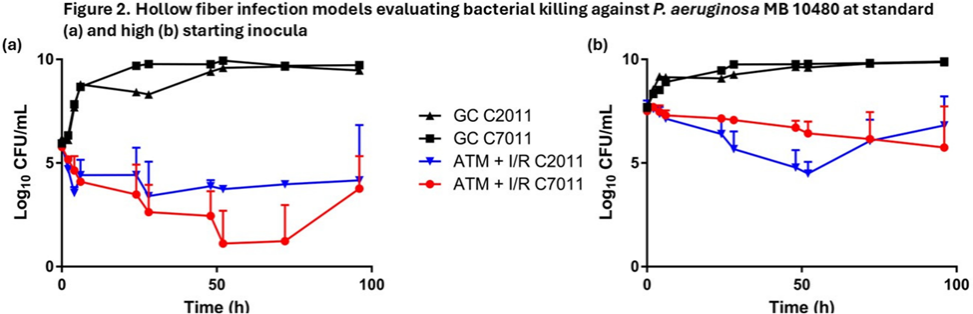
Hollow fiber infection models evaluating bacterial killing against P. aeruginosa MB 10480 at standard (a) and high (b) starting inocula ATM, aztreonam; I/R, imipenem/relebactam

**Methods:**

HFIMs using previously described PSA isolates [CL 5701 (constitutive PDC producer, OprD deleted) and MB 14080 (daughter isolate, IMP-1-producer)] were performed. Simulated exposures of the FDA-approved I/R regimen (0.5/0.5/0.25g q6h, 0.5 h infusion) was evaluated against CL 5701 while I/R + ATM (2g q6h, 2 h infusion) was evaluated against MB 14080. Each regimen was tested in duplicate at starting inocula of 6 log_10_ cfu/mL (standard) and 7.5 log_10_ cfu/mL (high). Each set of HFIM experiments was performed using paired C2011 and C7011 cartridges (same syringe, Duet^TM^, and peristaltic pumps for each pair). Changes in bacterial density from baseline were evaluated at hours 24 and 96.

**Results:**

For CL 5701, bacterial killing was limited in the C2011 HFIM at the standard inoculum HFIM studies and regrowth observed after 24 hours. In contrast, bactericidal activity was observed at hours 24 and 96 (-3.53 and -4.55 log_10_ cfu/mL, respectively) with C7011. In the high inoculum HFIM of CL 5701, no notable differences in killing were observed between cartridges at hours 24 (C2011:-0.74 and C7011: -1.26 log_10_ cfu/mL) and 96 (-1.05 vs -0.85 log_10_ cfu/mL). For HFIM of MB10480 at the standard inoculum, bacterial killing was greater in C7011 relative to C2011 at hours 24 (-1.46 vs -2.29 log_10_ cfu/mL) and 96 (-1.72 vs -2.00 log_10_ cfu/mL). In high inoculum MB 10480 HFIM studies, killing was greater in C2011 relative to C7011 at hour 24 (-1.27 vs -0.35 log_10_ cfu/mL), however there was more pronounced killing and less regrowth with C7011 vs C2011 at hour 96 (-0.85 vs -1.76 log_10_ cfu/mL).

**Conclusion:**

Differences in bacterial killing were apparent when comparing the effect of I/R and I/R+ATM in HFIM of PSA with different infection cartridges, likely due to more β-lactamase retention in less porous standard cartridges (C2011).

**Disclosures:**

J Nicholas O'Donnell, Pharm.D., MSc, Merck and Co., Inc: Grant/Research Support Nicole L. Shakerley, PhD, Merck and Co., Inc: Grant/Research Support Katherine Young, M.S., Merck & Co., Inc.: Stocks/Bonds (Public Company) Thomas Lodise, Jr., PharmD, PhD, GSK: Advisor/Consultant

